# Occurrence and spread of influenza A(H1N1)pdm09 virus infection in Norwegian pig herds based on active serosurveillance from 2010 to 2014

**DOI:** 10.1017/S0950268816001424

**Published:** 2016-07-14

**Authors:** C. ER, E. SKJERVE, E. BRUN, T. FRAMSTAD, B. LIUM

**Affiliations:** 1 Norwegian Veterinary Institute, Oslo, Norway; 2 Norwegian University of Life Sciences, Campus Adamstuen, Oslo, Norway

**Keywords:** Active serosurveillance, influenza A, pandemic H1N1, pig, temporal and spatial

## Abstract

The incursion of influenza A(H1N1)pdm09 virus was detected by Norway's active serosurveillance of its pig population in 2009. Since then, surveillance data from 2010 to 2014 revealed that 54% of 5643 herd tests involving 1567 pig herds and 28% of 23 036 blood samples screened positive for antibodies against influenza A virus. Positive herds were confirmed to have influenza A(H1N1)pdm09 virus infection by haemagglutination inhibition test. In 50% of positive herd tests, ⩾60% of the sampled pigs in each herd had antibodies against influenza A(H1N1)pdm09 virus. This within-herd animal seroprevalence did not vary for type of production, herd size or year of test. The overall running mean of national herd seroprevalence, and annual herd incidence risks fluctuated narrowly around the means of 45% and 32%, respectively, with the highest levels recorded in the three densest pig-producing counties. The probability of a herd being seropositive varied in the five production classes, which were sow pools, multiplier herds, conventional sow herds, nucleus herds, and fattening herds in descending order of likelihood. Large herds were more likely to be seropositive. Seropositive herds were highly likely to be seropositive the following year. The study shows that influenza A(H1N1)pdm09 virus is established in the Norwegian pig population with recurrent and new herd infections every year with the national herd seroprevalence in 2014 hovering at around 43% (95% confidence interval 40–46%).

## INTRODUCTION

Influenza A viruses (IAVs) are ubiquitous in both humans and animals, and are endemic in most pig populations worldwide [1–6]. Several short-term influenza virus surveillance systems in the last two decades [7–13] revealed that the dominant circulating swine influenza A viruses (swIAVs) in European pigs were: the Eurasian avian-like H1N1 [14], human-like H3N2 [15], and triple assortant (swine, human, avian) H1N2 [4]. The most recent virus being influenza A(H1N1)pdm09 virus (H1N1pdm09), which joined the ranks of the preceding three subtypes with increasing incidence from 2010 [13, 16]. Subtype H1N1pdm09 was first reported in humans in April 2009, in North and South America [17]. Following outbreaks in humans, pig-producing countries worldwide increased their surveillance activities and also reported the detection of H1N1pdm09 in their pig populations [1, 18–20].

**Fig. 1.**
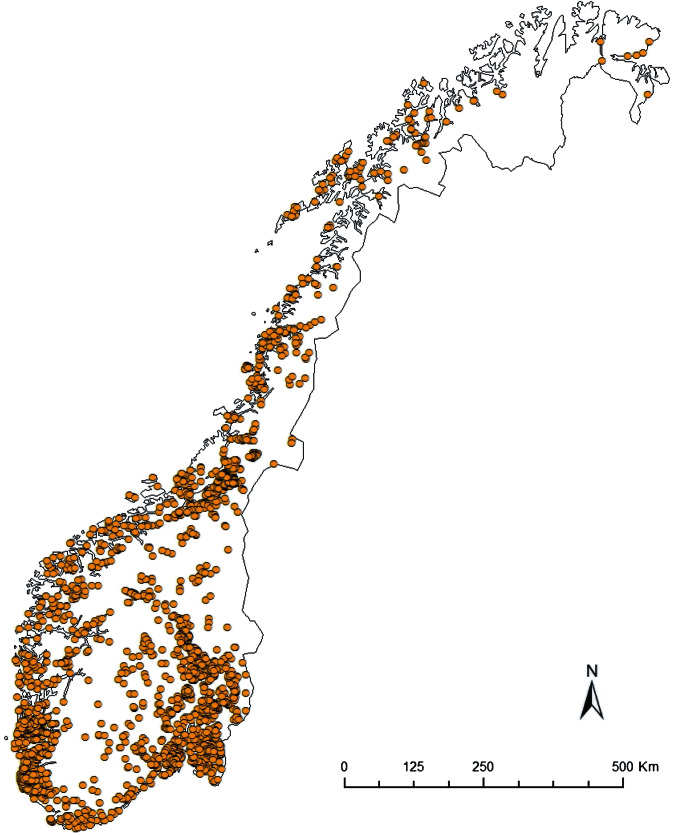
Spatial distribution of pig herds (*n* ≈ 2000) registered in Norway in 2014 [25].

However, such coordinated surveillance activities in pig populations were short term and on an *ad hoc* basis, usually undertaken when funding was available. While prominent organizations like the Centers for Disease Control and Prevention (CDC), the European Influenza Surveillance Network (EISN) and the World Health Organization (WHO) have well-developed and continuous human influenza surveillance systems [21], sustained influenza virus surveillance in pigs is absent in most countries because swine influenza typically is neither a reportable nor a regulated pig disease. Although influenza surveillance in pigs since the emergence of H1N1pdm09 has improved around the world, including Europe [16], surveillance of IAV in pigs remains passive for the most part [22, 23]. The major shortcoming of a passive surveillance system is that infections like H1N1pdm09 in pigs can pose a problem because subclinical cases are often missed. A case-control study involving 118 nucleus and multiplier herds in Norway showed that only 19 (40%) of 48 seropositive herds had detectable clinical signs [24]. As such, the study of prevalence, incidence risks and temporal trends for a largely subclinical infection like H1N1pdm09 is difficult under passive surveillance systems. Herd prevalence, incidence and temporal trends of H1N1pdm09 infection in pigs, could, however, be studied in depth in Norway because swine influenza is a reportable disease and vaccination of pigs against swIAV is not practised. From the ~2000 pig herds in Norway (Fig. 1), about one third (500–750) of the herds are selected every year for screening against IAVs and other reportable diseases [25].

The ongoing annual active serosurveillance of swIAVs in Norway that began in 1997 [26], more than a decade before the outbreak of H1N1pdm09 infection in pigs, ascertained that its pig population had been free from all IAVs prior to the incursion of H1N1pdm09 in October 2009 [18]. This occurred a few months after the first human cases caused by the same strain of influenza virus were diagnosed in Norway [18, 27]. The same case-control study on clinical impact of the infection mentioned earlier also revealed that infected humans had transmitted the virus to the pigs by reverse zoonosis while working in close proximity with the pigs [28]. A ramped-up risk-based surveillance, following diagnosis of the index case herd, discovered that the infection had quickly become widespread in pig herds throughout Norway [27]. Ninety-one out of 215 herds tested positive serologically or by PCR testing within a 3- month period. The simultaneous detection in so many pig herds dispersed across Norway in a short time suggested that the incursion was not a point-source pattern that is typical of diseases spread by animal movements and animal contacts. The initial planned eradication of the virus from the pig population by depopulation was aborted because it was deemed ineffective and cost-prohibitive. In addition, the prospect of humans as continued potential sources of infection to pigs also discouraged eradication procedures. The common view at the time was that the infection was expected to burn out with time given its highly contagious nature, short incubation, quick infective phase and recovery, especially in the relatively small pig herds typical in Norway [29]. Five years after the incursion in 2009, this has not happened. In 2014, the national herd seroprevalence remained high at >40% [25].

The accumulated data collected from Norway's ongoing active national serosurveillance of H1N1pdm09 virus gave us the opportunity to study the ecology of the virus, and the natural progression and epidemiology of this infection in the Norwegian pig population, which was a formerly naive population for all IAVs.

## MATERIALS AND METHODS

**Fig. 2.**
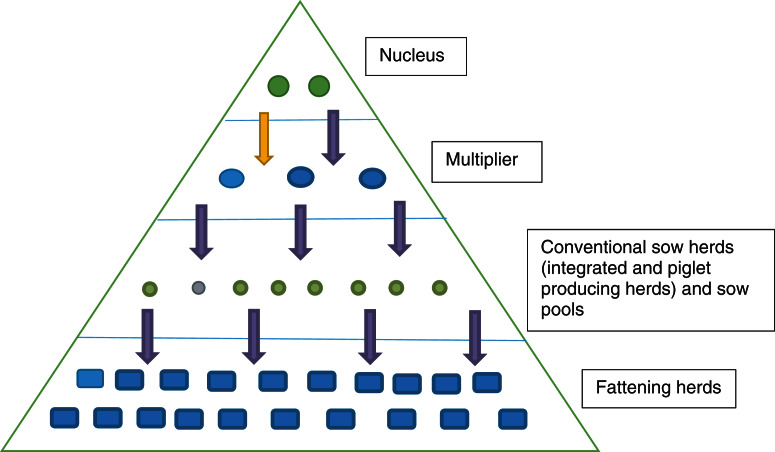
Pyramid system of Norway's pig production system showing a unidirectional flow to optimize health and performance of genetic lines and heterosis.

The 5-year surveillance data from 1 January 2010 to 31 December 2014 involved 1567 pig herds (~75% of the 2000 pig herds in Norway based on the National Registry of Pig Herds, 2014) with a total of 5643 herd tests and a total of 23 026 individual blood samples (Fig. 1). Pig herds in the sampled population were classified into five production classes: (1) fattening; (2) nucleus herds; (3) multiplier herds; (4) conventional sow herds, and (5) sow pools. These five classes of pig herds form the breeding and health pyramid that creates a unidirectional animal flow in the production of pig meat (Fig. 2). At the top are the closed nucleus herds (*n* ≈ 40) where pure genetic lines are constantly improved. Expanding in the next level are the multiplier herds (*n* ≈ 60) where some multiplier herds are closed and most are associated with one nucleus herd. They produce maternal lines of Landrace-Yorkshire (LY) cross and supply gilts to conventional sow herds, which include both integrated and piglet-producing herds. Nevertheless, some commercial sow herds do replenish their sow numbers with gilts from their own production. Unique to Scandinavian countries with their small sow herds, the sow pool system in Norway involves a cooperation between 10–20 pig producers where one central gestation herd supplies the cooperating producers (satellite units) with pregnant sows in a leasing system [30, 31]. Tables 1 and 2 give a breakdown of the number of herd tests by the five production classes in the 19 counties of Norway.

**Table 1.** Active serosurveillance for influenza A virus infection in the Norwegian pig population. Number of herd tests by county (n = 19) from 2010 to 2014

County	2010	2011*	2012	2013	2014	Total
Østfold	44	89	95	95	50	373
Akershus/Oslo	27	79	59	47	36	248
Hedmark	41	126	152	131	120	570
Oppland	31	109	94	102	69	405
Buskerud	8	37	44	41	24	154
Vestfold	37	84	91	79	66	357
Telemark	8	28	29	25	28	118
Aust-Agder	2	5	1	6	6	20
Vest-Agder	4	17	12	9	6	48
Rogaland	145	289	338	424	317	1513
Hordaland	15	24	26	34	20	119
Sogn og Fjordane	14	6	27	27	23	97
Møre og Romsdal	4	11	21	20	20	76
Sør-Trøndelag	14	52	44	57	50	217
Nord-Trøndelag	69	291	281	212	196	1049
Nordland	22	40	44	72	61	239
Troms/Finnmark	5	1	3	20	11	40
Total	490	1288	1361	1401	1103	5643

* A change in sampling strategy beginning in 2011 where the same herd could be tested more than once in the same year. Sampling took place at the slaughterhouses.

**Table 2.** Number of herd tests involving serosurveillance of influenza A virus infection classified by the five production classes from 2010 to 2014

Production class	2010	2011*	2012	2013	2014	Total
Fattening herd	50	79	64	65	47	305
Nucleus herd	52	79	78	82	71	362
Multiplier herd	73	117	152	148	137	627
Conv. sow herd	302	950	996	1047	798	4093
Sow pool	13	63	71	59	50	256
Total	490	1288	1361	1401	1103	5643

* A change in sampling strategy beginning in 2011 where the same herd could be tested more than once in the same year. Sampling took place at the slaughterhouses.

### Herd sampling

The Food Safety Authority carries out the sampling based on herds selected by the Norwegian Veterinary Institute every year. All nucleus, multiplier and sow pool herds are tested every year because they are high priority herds. Blood samples of ten pigs from all nucleus and multiplier herds (*n* = 97 in 2014) as well as 30 blood samples from the gestation units of every sow pool (*n* = 14 in 2014) are sampled annually from each herd. Prior to 2011, conventional sow herds were proportionally selected annually from each of the 19 counties according to the number of herds registered with the National Registry of Pig Herds. In each of these herds, blood samples were taken from ten sows. However, in 2011 there was a change in the sampling strategy for conventional sow herds in that blood samples are now collected from slaughtered sows and boars at the 12 largest abattoirs where more than 97% of the pigs in Norway are slaughtered. The number of blood samples collected at each slaughterhouse per year is proportional to the total number of adult pigs slaughtered per year. Sampling days are distributed evenly throughout the year. Blood samples are collected from one to five sows for each selected herd and the same herd could be sampled several times a year. Regarding fattening herds, ten blood samples are collected every year from 40–60 selected herds.

With such a mixed sampling design, the number of pigs sampled per herd test is non-uniform across the five production classes. Overall in our study, there were 3562 (63%) herd tests with <5 pigs sampled. In proportion to each production class with such small size samples, conventional sow herds had the highest with 77%, followed by multiplier herds with 38%, and nucleus herds with 30%. Of the 19 pig-producing counties of Norway, Rogaland, Nord-Trøndelag, and Hedmark with the largest number of pig herds, respectively, had the highest number of herd tests every year (Table 1).

### Laboratory analyses and herd diagnosis

All serological analyses were performed at the Norwegian Veterinary Institute in Oslo. A commercial competitive ELISA (ID Screen^®^ Influenza A Antibody Competition multi-species kit; ID VET, France) with a reported sensitivity of 93% and specificity of 99% (manufacturer's data) was the screening test for serum antibodies against IAV. The ELISA test can detect IAV antibodies in any species including pigs. Titres *⩾*40 were considered positive for IAV antibodies. In cases of positive or inconclusive results, the serum samples were re-tested using the haemagglutination inhibition test (HI), to detect antibodies against the four antigens, namely H1N1pdm09 (A/California/07/2009), European H1N1 [A/Sw/Belgium/1/98 (H1N1)], H1N2 [A/Sw/Gent/7623/99(H1N2)] and H3N2 [A/Sw/Flanders/1/98(H3N2)]. CDC identified and described the first antigen [32], while the latter three antigens were identified and described in Belgium [33]. Testing of these serotypes have been described in the *OIE Manual of Diagnostic Tests and Vaccines for Terrestrial Animals*. A herd was considered positive if at least one blood sample serially tested positive with ELISA first, followed by an HI test using antigens produced at the Norwegian Veterinary Institute. Pigs with an antibody titre ⩾10 in the HI test for a given subtype were regarded as positive or considered a cross-reaction if more than one type of antigen reacted positively. The herd-level diagnosis was based on which subtype had the highest mean titre, and the highest prevalence in a single herd test. Antigen reactions other than H1N1pdm09 were considered cross-reactions because they were either lower in titre, fewer in proportion in positive reactions, and unlike H1N1pdm09, they did not exist as single antigen reactions in any of the blood samples examined.

### Test sensitivity and specificity at herd level

Based upon the individual test sensitivity, we calculated herd sensitivity by two formulae:
1
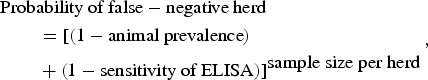

2
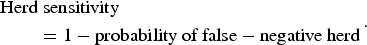


The calculations show that a sample size of ten pigs per herd was sufficient to achieve at least 95% confidence of identifying a positive herd based on a within-herd prevalence of 26%. As the number of pigs sampled per herd test varied considerably (range 1–40 pigs) and the animal prevalence also varied between herds, some herd tests were lower in sensitivity, and some higher. The probability of falsely classifying a positive herd as negative therefore is higher in herds with few pigs sampled and lower animal prevalence. Conversely, it is harder to classify a negative herd as falsely positive because all positive tests were followed by a HI test. Serial testing raised the specificity to almost 100% at the herd level.

### Temporal and spatial analysis

Temporal trends of herd seroprevalence were investigated using Stata's lowess smoothing function (Stata v. 14.0, StataCorp LP, USA) to plot the running means of the herd infection status (seropositive = 1, negative = 0) against the sampling dates from 2010 to 2014. Stratifying herd seroprevalence by the 19 counties enabled the investigation of spatial variations across Norway with varying pig-farming densities in different counties. Stratifying the seroprevalence by the five production classes allowed the investigations of variations in probability of infection between the five different types of farm operation. The uniform distribution of herd sampling over 12 months enabled us to use day as the time unit to plot the temporal trends. The lowess smoothing function examined the spatial correlations between the probabilities of herds being seropositive with pig-farming density by plotting the mean herd seroprevalence with the mean distance of the four nearest pig herds. The mean distance of the four nearest pig herds was a proxy indicator of pig herd density.

### Environmental and production conditions

To identify production factors associated with a seropositive pig herd, we used Stata to execute a mixed logistic regression analysis on the hierarchical data (*N*_county_ = 19, *N*_herds_ = 1567, *N*_herd tests_ = 5643) for the binary outcome of a herd testing positive.

With the existing sampling plan, each herd could have been sampled multiple times (between 1 and 12 times per year and up to 29 times during the 5-year study period). The data were nested in herd identity and county and were thus included as random effects in our logistic regression model. The random effects account for all variances of non-fixed effects related to the county and the individual herd to give as accurate as possible the estimates on the fixed effects.

Herd size, a continuous variable, was based on the live pigs on each farm as reported by pig farmers to the National Registry of Pig Herds twice a year on 31 July and 31 December. Given that herd size had a nonlinear relationship with outcome, the herd size data were transformed into an ordinal variable with three quantiles using specific cut-off points of pig numbers to give small (<350 pigs, *n* = 698), medium (351–665 pigs, *n* = 450), and large (666–4075 pigs, *n* = 419) herds.

Presence of sows and being a closed or open herd were collinear with production class and hence were excluded from the model. Similarly, the mean distance from the four nearest herds was highly correlated with the 19 counties and was therefore excluded as a predictor from the final mixed regression model.

The three categorical fixed effects in the mixed logistic regression model were:

*Year of test (n = 5)*: included five years (2010, 2011, 2012, 2013, and 2014). *Herd size (n = 3)*: included three categories of small, medium and large. *Production class (n = 5)*: consisted of fattening herds, nucleus herds, multiplier herds, conventional sow herds and sow pools.

The mixed random-intercept logistic regression models were formulated as follows:

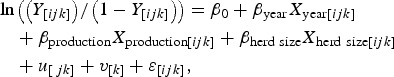

where *Y*_*ijk*_ is the binary outcome, where 0 = negative, 1 = positive of a herd test for the *i*th observation (*i* = 1, 2, …, 5643), *j*th herd (*n*_*j*_ = 1567) nested within the *k*th (*n*_*k*_ = 19) county; *β* is a vector of coefficients for the three categorical fixed effects: (1) year of test, (2) herd size, and (3) production class; *X*[_*ijk*_] is the vector of for the three predictors in our two models: (1) year of test, (2) herd size, and (3) production class for the *i*th observation of the *j*th herd and *k*th county; *u*_*jk*_ is a vector of random intercepts unique to each herd, where *u*_*jk*_ ~ *N* (0, *σ*^2^_herd_), and *v*_*k*_ is a vector of random intercepts unique to each county, where *v*_*k*_ ~ *N* (0, *σ*^2^_county_); and *ε*_*ijk*_ is the vector of error terms where *ε*_*ijk*_ ~ *N*(*μ, σ*^2^).

The likelihood ratio test aided model selection. To decide on the significance of additional predictors for the two models, a difference of <2 of the log likelihood score was regarded as non-significant and the most parsimonious model was chosen [34]. We tested the models by assessing fit and residual patterns.

There were 1816 herd tests where herds were tested consecutively for at least 2 years. To examine recurrent herd infection rates, univariable logistic regressions stratified on production class estimated the probability that a seropositive herd would be seropositive again the following year. Similarly, we investigated new herd infection rates by estimating the probability that a seronegative herd would test seropositive the following year by using mixed logistic regression.

We also plotted the incidence risks stratified by the four production classes of fattening, nucleus, multiplier and conventional sow herds. We excluded the sow pools from this analysis given their small numbers of only 14 herds and also that there was only one or no uninfected sow pool herds to calculate incidence risk for the following year.

### Within-herd seroprevalence

We investigated the within-herd animal seroprevalence by observing the proportion of pigs testing positive in 1028 positive herd tests that had at least five pigs tested. A cumulative probability on animal prevalence plot of these positive herds revealed the infectiveness of the disease in pigs kept in close proximity, typical of pig production. Factors causing variations to animal prevalence were investigated with scatter plots for the three categorical fixed effects of interest, i.e. year of test, production class and herd size. Graphical analyses were followed up with multivariable regression using general linear regression for categorical variables to investigate whether within-herd prevalence varied with the same three fixed effects.

Confidence intervals (CIs) using Stata for binomial outcomes gave inferential statistics on binomial probabilities of prevalence and incidence [35].

## RESULTS

### Herd seroprevalence, temporal trends

Surveillance data of 5643 herd tests on 23 039 samples from 2010 to 2014 showed that 6513 (28%) of the samples screened ELISA positive for antibodies against IAV in 2470 herd tests. Of these blood samples positive for antibodies against IAV, 5857 were confirmed by the HI test to be antibodies against H1N1pdm09 with 23·6% showing reactions to sole antigen H1N1pdm09. Seventy-six per cent of the samples with reactions to multiple antigens in addition to H1N1pdm09 were all deemed cross-reactions by our criteria for herd diagnosis.

**Fig. 3.**
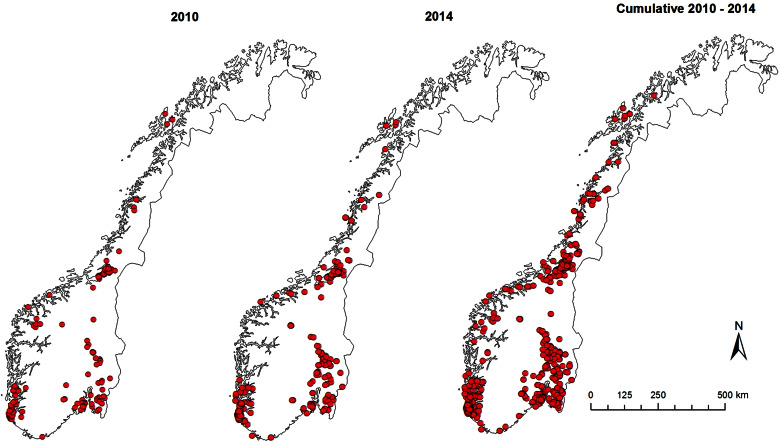
Spatial distribution of Norwegian pig herds testing positive for antibodies against influenza A(H1N1)pdm09 virus in 2010 [41%, 95% confidence interval (CI) 37–45 seroprevalence]; 2014 (48%, 95% CI 45–51 herd seroprevalence); cumulative 2010–2014 (53%, 95% CI 50–56 herd seroprevalence).

**Fig. 4.**
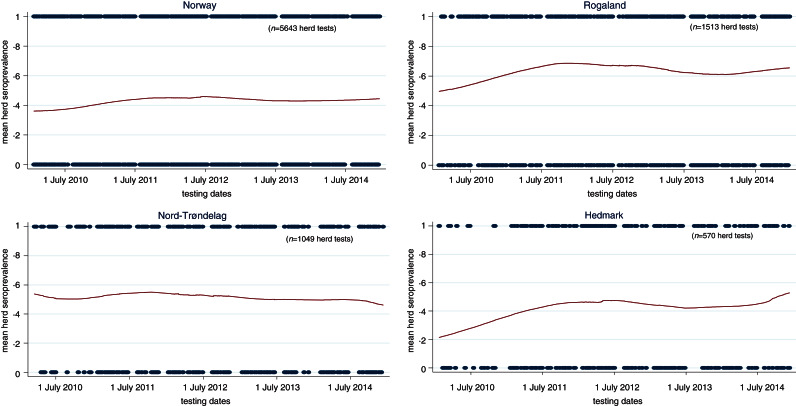
Temporal trends* of pig herd seroprevalence for influenza A(H1N1)pdm09 virus infection in Norway, stratified by top three pig-farming counties from 1 January 2010 to 31 December 2014. (* Using Stata's lowess smoothing plots to show running mean between positive herds and negative herds. Date is the unit measure for cross-section of proportion of positive herds.)

**Fig. 5.**
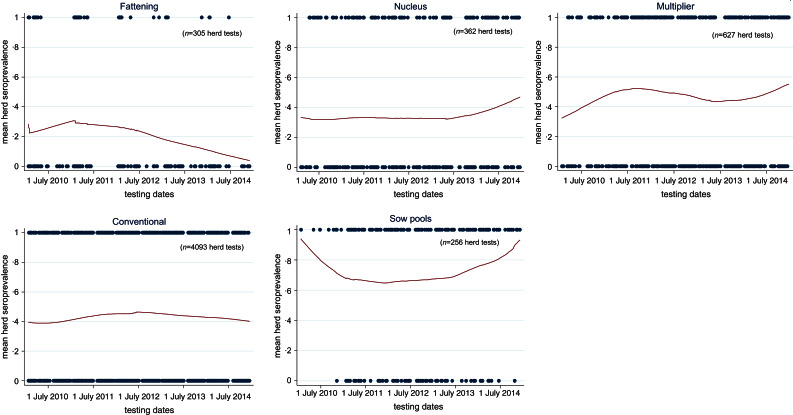
Temporal trends* pig herds seroprevalence of influenza A(H1N1)pdm09 virus infection in Norway, stratified by five production classes from 1 January 2010 to 31 December 2014. (* Using Stata's lowess smoothing plots to show running mean between positive herds and negative herds with date as the unit measure for cross-section of proportion of positive herds.)

Figure 3 shows the spatial distributions of seropositive herds in 2010, 2014 and cumulatively from 2010 to 2014. Of the 1567 herds involved in the surveillance, 842 tested positive at least once thus giving a national cumulative herd seroprevalence of 54% (95% CI 51–56) by the end of 2014. There were no unique clustering patterns for positive herds. The heavy pig-farming-area counties correspondingly also had higher herd prevalence. As depicted by the temporal and spatial trends in Figure 4, the running mean herd seroprevalence for the top three major pig-producing counties, Rogaland, Nord-Trøndelag, and Hedmark fluctuated between 20% and 70% with no signs of decreasing at the end of 5 years after incursion of the virus in 2009. Nationally, the trajectory of the running mean herd prevalence was flat and hovered at around 42%. Comparison of the temporal trends of herd seroprevalence in the five production classes seen in Figure 5 revealed that fattening herds had the lowest running herd mean seroprevalence, which rose from ~20% in 2010 to 30% in 2011 before gradually decreasing to 9% in 2014. The three production classes of nucleus, multiplier and conventional sow herds had similar trajectories of running mean herd seroprevalence that fluctuated between 40% and 50%. In contrast to the fattening herds, the small group of sow pool herds had the highest levels of mean herd seroprevalence. Depicted by a wide U-shaped trajectory in Figure 5, sow pool herds began with nearly 100% seroprevalence in 2010, which fell to ~62% in 2011 before gradually rising to ~90% in 2014.

### Recurrent herd infections

Looking at herds that were consecutively tested, there were 293 sow herds (nucleus, *n* = 41; multiplier, *n* = 51; conventional, *n* = 188; sow pools, n = 13) that were tested for multiple 4 or 5 years. The proportion of these herds by production class that were repeatedly seropositive for 4 or 5 years, were 11/41 (27%) for nucleus herds, 20/51 (40%) for multiplier herds, 54/188 (29%) for conventional sow herds and 11/13 (85%) for sow pools. Conversely, the proportion of herds that tested negative for all the years they were tested were 11/41 (27%) for nucleus herds, 7/51 (14%) for multiplier herds, 23/188 (12%) for conventional sow herds and 0/13 (0%) for sow pools.

### Herd incidence risk, temporal trends

**Fig. 6.**
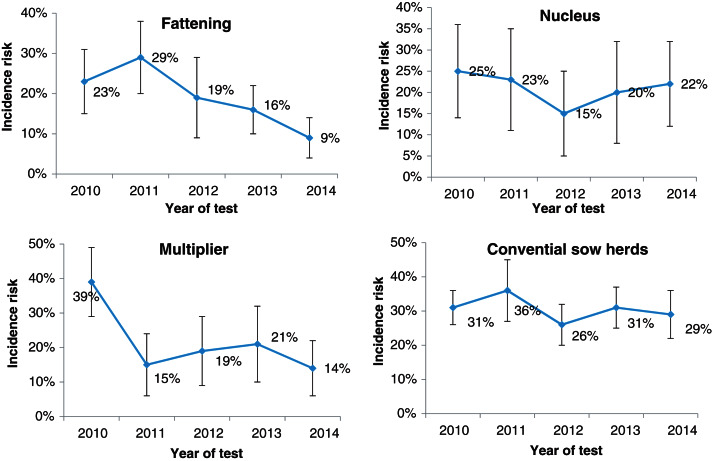
Estimates of incidence risks with 95% confidence intervals of new pig herd infections of influenza A(H1N1)pdm09 virus infection in Norway stratified by four production classes from 1 January 2010 to 31 December 2014

Figure 6 shows herd incidence risks or the proportion of new infections by production classes plotted over the 5 years. Temporal trends of incidence risks combined with recurrent infection trends (not shown) would give our seroprevalence trends in Figure 4. Incidence risks of fattening herds rose from ~23% in 2010 to 29% in 2011 before trending downwards towards 9%. Multiplier herds had a sharp drop from ~39% in 2010 to 15% in 2011, where it remained in the range between 14% and 21%. Nucleus herds had a V-shaped pattern where their incidence risk dropped from ~25% in 2010 to 15% in 2012 before rising to 22% in 2014. Conventional sow herds fluctuated between 26% and 36%, which were potentially the most underestimated in the production classes because of the low sample sizes associated with these herds.

### Herd prevalence and pig-farming density

**Fig. 7.**
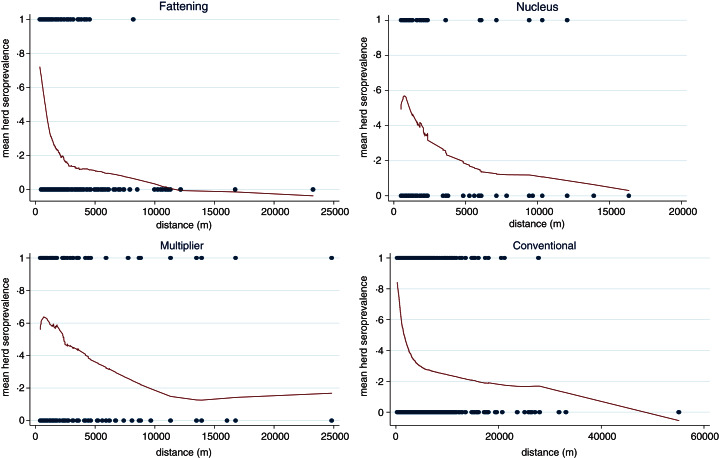
Lowess smoothing curves showing spatial relationship of running mean herd seroprevalence with mean distance of four nearest pig herds.

The lowess smoothing plots in Figure 7 show that the mean running herd seroprevalence of the four production classes (fattening, nucleus, multiplier and conventional herds) were inversely proportional to the mean distance of the four nearest pig herds. We omitted sow pools because of the low numbers involved.

### Within-herd prevalence

**Fig. 8.**
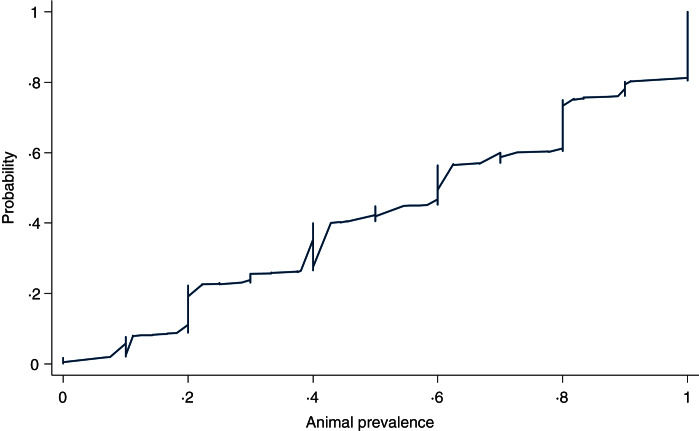
Cumulative probability of the proportion of pigs screened ELISA-positive for influenza A antibodies in herds (*n* = 1028) diagnosed positive for influenza A(H1N1)pdm09 with at least five pigs sampled.

Figure 8 shows a cumulative probability plot of the proportion of samples that tested positive in herds with at least five animals sampled (*n* = 1028 herd tests in 488 herds). The 10th percentile was 20%, 25th percentile, 30%, 50th percentile or median, 60%, 75th percentile 81%, 90th percentile 100%. We did not find any variations in animal prevalence in the five production classes or in the three quantiles of herd sizes using graphical comparisons (not shown) or multivariable regression analysis (not shown).

### Sensitivity of herd test

Given the possible variations in animal prevalence as shown in Figure 8 and the varying number of pigs per herd test, Table 3 shows how the sensitivity of the herd test varied with the number of pigs sampled for a herd test. Given the bulk (72%) of the herd tests came from conventional sow herds and the bulk of these tests involved only one pig, the chance of misclassifying a positive herd as negative is >44% likely in 50% of true positive cases (based on median in Table 3).

**Table 3.** Sensitivity of herd test with respect to animal prevalence and number of pigs sampled per herd test

No. of pigs sampled	Animal prevalence (percentile)				
	20% (10th)	30%(25th)	60% (median)	81% (75th)	100% (90th)
1	13	23	53	74	93
2	24	41	78	93	100
3	34	54	90	98	100
4	43	65	95	100	100
5	50	73	98	100	100
6	57	79	99	100	100
7	62	84	99	100	100
8	67	88	100	100	100
9	71	90	100	100	100
10	75	93	100	100	100

Values given are percentages.

### Unconditional mixed logistic regression model

Mixed logistic regression analysis (Table 4) shows the herd was equally likely to be tested positive in any of the 5 years. Positive herds were either recurrent cases or new herd cases depicted by our incidence plots in Figure 6. The medium and large herds were more likely than the small herds to be seropositive, while the difference between medium and large herds were not significant. In terms of production class, fattening herds had the lowest probability while sow pools had the highest with an odds ratio (OR) of 24. The other three production classes of nucleus, multiplier and conventional sow herds had ORs of 2·78, 4·72 and 2·63, respectively.

**Table 4.** Mixed logistic regression of the binomial outcome that a herd test was positive for antibodies against influenza A(H1N1)pdm09 virus infection based on haemagglutination inhibition test

No. of observations (positives)	Fixed effects*	OR	95% CI	*P* value
	Year of test (*n* = 5)			
490 (204)	2010	1		
1288 (563)	2011	1·02	0·78–1·33	0·89
1361 (641)	2012	1·07	0·82–1·39	0·62
1401 (587)	2013	0·82	0·64–1·07	0·14
1103 (478)	2014	0·88	0·67–1·15	0·35
	Production class (*n* = 5)			
305 (69)	Fattening herd	1		
362 (123)	Nucleus herd	2·78	1·67–4·62	<0·001
627 (297)	Multiplier herd	4·72	3·05–7·30	<0·001
4093 (1826)	Conventional herd	2·63	1·87–3·70	<0·001
256 (155)	Sow pool	24·04	10·98–52·66	<0·001
	Herd size in three quantiles (pigs)			
1679 (601)	Small (<350 pigs)	1		
1909 (919)	Medium (350–665 pigs)	1·85	1·52–2·26	
1851 (881)	Large (>665 pigs)	1·63	1·33–1·99	
	Constant	0·50	0·33–0·75	
	Random effects†			
5643 (2473)	County (*n* = 19)			
	Var(const.)	4·45	2·16–9·15	
5643 (2473)	County>herd id (*n* = 1567)			
	Var(const.)	1·66	1·29–2·15	

OR, Odds ratio; CI, confidence interval.

* Three categorical fixed effects were: (1) year of test, (2) production class, and (3) herd size (based on national registry for subsidy).

† County and herd ID were included as random effects to account for non-fixed effects associated with county and the individual herd.

For herds (*n* = 1327) that were negative and tested again the following year, Table 5 shows the results of a mixed logistic regression analysis of the probability of new infection (incidence risk). The risk of a new herd infection progressively increased from 2010 to 2014. With an OR of 48, multiplier herds were most likely to be newly infected. The risk in conventional sow herds (OR 9·75) was closer to the nucleus herds (OR 6·26). The risk of being infected were also stepwise higher with medium-sized herds having an OR of 6·3 and large herds having an OR of 9·2. Sow pool herds were dropped from the analysis because of small numbers (*n* = 14) and that they were either all positive and therefore had predicted the following year perfectly or there was one negative sow pool herd that was predicted perfectly to be negative next year.

**Table 5.** Mixed logistic regression on the binomial outcome that a negative herd would test positive the following year for antibodies against influenza A(H1N1)pdm09 using the haemagglutination inhibition test

Number of observations (positives)	Fixed effects*	OR	95% CI	*P* value
	Year of test (*n* = 5)			
174 (46)	2010	1		
406 (104)	2011	1·30	0·55–3·05	0·553
358 (121)	2012	5·72	2·31–14·17	<0·001
389 (128)	2013	6·40	2·43–16·83	<0·001
	Production class (*n* = 4)			
258 (64)	Fattening herd	1		
157 (43)	Nucleus herd	6·26	1·12–34·86	0·036
155 (56)	Multiplier herd	47·95	7·75–296·83	<0·001
757 (236)	Conventional herd	9·75	2·85–33·41	<0·001
	Herd size in three quantiles (pigs)			
399 (89)	Small (<350)	1		
405 (130)	Medium (350–665)	6·31	2·00–19·87	0·002
469 (165)	Large (>665)	9·24	2·78–30·72	<0·001
	Constant	0·0004	0·00003–0·00612	<0·001
	Random effects†			
1327 (399)	County (*n* = 17)			
	Var(constant)	2·72	1·01–7·35	
1327 (399)	County>Herd id (*n* = 621)			
	Var (constant)	19·72	10·34–37·61	

OR, Odds ratio; CI, confidence interval.

* The three categorical fixed effects were (1) year of test, (2) production class, and (3) herd size.

† County and herd ID were included as random effects to account for non-fixed efects associated with county and the individual herd.

## DISCUSSION

What began in October 2009 as Norway's first IAV infection in pigs, H1N1pdm09, spread rapidly to naive pig herds throughout the country [18, 27]. The initial herd seroprevalence at the end of 2009 was 18%. In the absence of any vaccination practice, intervention measures from the food safety authorities, and the absence of other swIAVs, the seroprevalence had climbed to >40% in 2010 and has remained >40% ever since. The consistently high herd seroprevalence of the virus over the years was due partly to sustained recurrent infections in positive herds with sows (ranging from the lowest 27% in nucleus herds to the highest of 85% in sow pool system) and partly to new herd infections as evidenced by the 9–39% incidence risks in the four productions classes (Fig. 6).

Considering the rapid turnover of sows in Norwegian sow herds (culling at average three parities or ~2 years old), positive sow herds that were still positive after 2 years were the result of recurrent infections. Recurrent infections in positive herds occur frequently in nucleus, multiplier and conventional sow herds but with highest probability in the smallest group, the sow pools. This is not surprising because of this group's unique pattern of frequent contacts between multiple satellite herds.

Spatial analysis revealed that the counties with the three densest pig populations also correspondingly had the highest proportion of positive herds (Fig. 4). This is unsurprising as pig production is characterized by animals kept in close proximity and high turnover rate leading to susceptible new hosts being produced rapidly as required by highly contagious pathogens like IAV to propagate and maintain itself in the population. Higher density pig-farming counties also mean larger quantities of virus shed into the environment which increases the probability of transmission to susceptible hosts. Other studies have also shown that higher pig-density areas also have higher rates of respiratory diseases [36, 37].

In Norway, the persistence of H1N1pdm09 in the pig population can be attributed to several production factors that favour the infection dynamics of the influenza virus. Although our study has not ascertained the sources of the virus in these recurrent and new infections, the varying production class-specific probabilities revealed in our regression analysis and graphical plots suggest transmission patterns are related to their production operations. Elsewhere, since the outbreak of H1N1pdm09 in pigs, there have been studies on pigs such as those by the EISN, investigating the dominant swIAV subtypes circulating in European pig populations [13], and also *ad hoc* surveillance studies conducted to investigate the persistence and transmission dynamics of influenza viruses circulating in some European pig herds (Belgium, France, Italy, Spain) [38, 39]. These studies indicated that although there were various swIAVs circulating, some pig farms continually tested positive for the same swIAV subtypes over the six sampling periods from 2006–2009. Persistence of infection from horizontal transfer between animal contacts within these herds or re-introduction due to poor biosecurity was put forward as possibilities for these herds repeatedly testing positive. Although the scale of these studies was much smaller (3–80 herds) and the scope was restricted to only farrow-to-finish herds, the results on the dynamics of pig-to-pig transmission are partly helpful in elucidating the patterns of recurrent infections and new herd infections seen in our study.

Previous studies have shown that people working with pig herds may have transmitted H1N1pdm09 to pigs [7, 12, 16, 18, 20, 28]. Here in Norway, reverse zoonosis of humans carrying the virus and infecting the pigs they are in contact with remained highly probable during the study period. National influenza virus surveillance in humans by the Norwegian public health authorities during the previous two influenza seasons from 2012 to 2014 shows that more than 50% of all human influenza cases in Norway were still caused by H1N1pdm09 [40]. Hence, right up to the end of 2014, spillovers from human infections could have been an important source of virus for recurrent or new herd infections, especially so for nucleus herds which are closed to the introduction of pigs from other herds. Fattening herds had the lowest levels of herd seroprevalence consistently for all 5 years compared to the other production classes. This would be surprising if animal contacts were the sole mode of transmission because the majority of fattening herds buy piglets from many herds without requiring documentation of freedom from H1N1pdm09. Many fattening herds are all-in/all-out operations, at least at room level, with no contact between batches of pigs. Even if the young growers (~30 kg) came from positive herds, maternal antibodies may have protected them from infection and kept them virus free for transfer to the fattening herd. In fattening herds, fewer close human–pig interactions may have also contributed to a lower herd seroprevalence/incidence compared to the other four production classes. We see in Figures 5 and 6 that the running herd prevalence and incidence risks of fattening herds showed a marked decline after peaking in 2011. An explanation for the decline could be that more piglet-producing sow herds had developed active immunity with time and consequently fortified fattening pigs with protective maternal antibodies crucial for protection during the vulnerable transition to the grower phase where mixing between new pigs occurs. Nevertheless, new herd infections in fattening herds every year were still occurring as evidenced by the non-zero incidence risks. It was also highly likely that carrier piglets in the batch of fattening pigs could become a source of infection to other pigs that would become susceptible when maternal antibodies waned sufficiently [39, 41, 42]. Age-related factors could play a role in causing the differential patterns in seroprevalence in the five production systems, especially between fattening herds and the other four production systems. Pigs sampled from fattening herds at the slaughterhouse were aged ~6 months whereas for other four types of sow herds (nucleus, multiplier, sow pools, conventional sow herds), older pigs like sows are sampled. Older animals, by virtue of their longer existence also means that their probabilities of exposure to the virus during their slightly extended lives before being sampled are higher.

The small group of sow pool herds (*n* = 14) had the highest levels of seroprevalence because they had the highest rates of recurrent infections. This was expected given their special operating mode that allows mixing of sows from various satellite pig herds (*n* = 10–20), thereby increasing the risk of horizontal spread between herds. Human–pig contact frequency and the accumulated duration with different people are also higher in the sow pool system thus increasing the sources and risk of reverse zoonosis.

Regarding the nucleus, multiplier and conventional sow herds, their temporal trends in mean herd seroprevalence did not differ much. These three types of production classes fluctuated within a narrow range between 40% and 50%, with no signs of abating towards the end of 2014. Multiplier herds had a higher probability (OR 4·72, 95% CI 3·1–7·3) of testing seropositive compared to fattening herds. Nucleus herds, closed to pigs from other sources, had a lower probability of being positive (OR 2·78, 95% CI 1·7–4·6). Seroprevalence and incidence risks of conventional sow herds (OR 2·63, 95% CI 1·8–3·7) were unexpectedly similar to nucleus herds. The anomaly is accounted for by the lower sensitivity in herd tests for conventional herds because fewer pigs are sampled per herd test in these herds. As shown in Table 3, the likelihood of misclassification increases with decreasing within-herd prevalence and sample size. Herd tests involving conventional sow herds having ⩽4 pigs were disproportionately high (77%). Many of these herd tests involved only one pig. Sampling only one pig and given within-herd prevalences of 20%, 30%, 60%, 81%, and 100% (following the percentiles in Table 3) would respectively give herd test sensitivities of 13%, 23%, 53%, 74% and 93%. Therefore, the gap between the conventional sow herds and nucleus herds could be much wider when we factor in misclassification bias. In the same light, the seroprevalence trends depicted in Figures 4 and 5 (conventional sow herds in particular) reflect an underestimation of true herd prevalence since conventional sow herds made up the majority (72%) of the 5643 herd tests.

With regard to the closed nucleus herds with highest biosecurity, the recurrent infections in positive nucleus herds were likely caused by circulation of the virus within the herd or caused by continual spillovers from human infections as mentioned earlier. Nucleus herds generally have higher gilt replacement rates (60–70% are first-parity sows) in overlapping batches. The shorter cycle and higher rates of replacements also means that more newly susceptible hosts become available faster for new infections and the propagation of the virus.

As to animal prevalence in positive herds, ~60% of positive herds with at least 5 pigs sampled had ⩾50% of the animals testing seropositive (Fig. 8). The high within-herd seroprevalence is consistent with other swine influenza strains in that infections in pig herds are highly contagious with a short incubation period that could reach 100% infection rate within a short time [1, 39]. The animal prevalence did not differ between the production classes which is unsurprising given that the stocking density for all five production classes are similar and therefore would experience similar transmission dynamics for contagious diseases transmitted through contact and aerosols between animals in close proximity [1, 29].

The temporal trends of seroprevalence, recurrent and new herd infection rates observed in our surveillance data from 2010 to 2014 suggest that H1N1pdm09 will remain in the Norwegian pig population for as long as humans or pigs, or both, act as reservoirs and continue to transmit the virus to susceptible pigs. The persistence of the virus in the pig population has economic consequences even though the infection in the naive Norwegian pigs was mostly subclinical [24, 27]. A longitudinal study showed that the infection reduced the growth performance of growing pigs by reducing their feed efficiency. Infected grower pigs consumed more feed and had a protracted production time to reach the same market weight compared to their uninfected counterparts [26, 43].

H1N1pdm09 in pigs is not only widespread across Western Europe, reassortant subtypes have already appeared, probably due to co-infections of H1N1pdm09 with established swIAVs [13]. It follows in the presence of established swIAVs, the prevalence and infection rates of H1N1pdm09 in pig herds in Western Europe were considerably lower [12, 16] compared to Norway. Conversely, H1N1pdm09 is likely to remain geographically a stable lineage in the Norwegian pig population given its continued sole existence as the only subtype circulating and coupled with Norway's continued closed-door policy on movement of live pigs across its borders. Nonetheless, the prospect of reassortment with human influenza could potentially occur if farmers with human IAVs introduce them to the pigs again, especially for subtypes like human H3N2 virus, for which antibodies to H1N1pdm09 do not offer cross-protection against (based on unpublished serology data at the Norwegian Veterinary Institute). To minimize such possibilities, it seems appropriate to continue with the recommendation that people working with pig herds should be immunized regularly with human influenza vaccines, and refrain from contacts with pigs if they have influenza-like symptoms.

In conclusion, although sampling for herd tests had varied across the pig herds and therefore made direct prevalence inferences somewhat challenging, we think that Norway's active surveillance gives a fairly representative picture of the natural infection dynamics of the virus in the Norwegian pig population given the absence of any intervention measures.

Five years after the incursion of the new influenza virus, H1N1pdm09, the prevalence of seropositive herds has not fallen below 40%. This strongly indicates that the virus has adapted well and established itself in the Norwegian pig population. To mount measures against the spread of influenza virus to new pig herds, and to break the chain of infection in infected herds, further research on the transmission dynamics of the H1N1pdm09 virus in the Norwegian pig population and economic analyses would give farmers and the food safety authorities guidance on feasible approaches.
